# Impact of diabetes mellitus type two on incidence and progression of Parkinson’s disease: a systematic review of longitudinal patient cohorts

**DOI:** 10.1007/s00702-025-02882-7

**Published:** 2025-01-23

**Authors:** Olga Stockmann, Lan Ye, Stephan Greten, David Chemodanow, Florian Wegner, Martin Klietz

**Affiliations:** Department of Neurology, Hannover Medical School, Carl-Neuberg-Straße 1, 30625 Hannover, Germany

**Keywords:** Parkinson’s disease (PD), Diabetes mellitus, Incidence, Disease progression, Biomarker

## Abstract

Parkinson’s disease (PD) is a chronic neurodegenerative disease of the elderly. Patients suffer from progressive motor and non-motor symptoms. Further, PD patients often present geriatric features like multimorbidity and polypharmacotherapy. A frequent comorbidity of PD patients is diabetes mellitus type two (T2DM). In the last decade growing evidence emerged on the impact of T2DM on PD. Of the present review was to analyze the impact of T2DM on PD incidence and progression in patient cohorts. A systematic review of the literature was performed via PubMed and Google Scholar. Studies on longitudinal PD patient cohorts with at least 10 patients per group were included. The diabetic state of the patient had to be determined. In total, 15 studies were analyzed for this review. According to most of the included studies T2DM increases the risk of developing PD significantly. Disease progression is augmented by T2DM both for motor and cognitive impairments. Some studies also point out a correlation of motor worsening and diabetic status measured by the serum HbA1c level. In relation to biomarkers, PD patients with diabetes have higher neurofilament light chain and Tau level but lower Amyloid beta level. T2DM seems to be a risk factor for the development and progression of PD. PD patients should be screened for T2DM and treatment should be initiated promptly. There is still a lack of knowledge about the molecular mechanisms leading to interactions of these diseases.

## Introduction

Parkinson’s disease (PD) is a frequent neurodegenerative disease of the elderly leading to motor and non-motor symptoms. The characterizing motor symptoms are rigidity, tremor at rest and bradykinesia. Regarding non-motor symptoms especially neuropsychiatric symptoms and dementia can be burdensome for patients and their caregivers (Eichel et al. 2022; Veith Sanches et al. 2024). With increasing age PD patients display geriatric features like multimorbidity and polypharmacotherapy for their diseases, which may result in critical drug safety (Klietz et al. 2019; Greten et al. 2021).

In the last decade growing evidence emerged that diabetes mellitus type two (T2DM) is a frequent comorbidity of PD patients resulting in worse neurological outcomes. While biological pathways are still not completely understood, there seems to be some impact of T2DM on PD incidence and progression (König and Outeiro 2024).

Aim of the present systematic review is to analyze longitudinal patient cohorts regarding T2DM as modulator of PD incidence, disease progression and changes in the biomarker profile. These data are needed for optimized treatment regiments.

## Methods

### Inclusion criteria

**Fig. 1:**
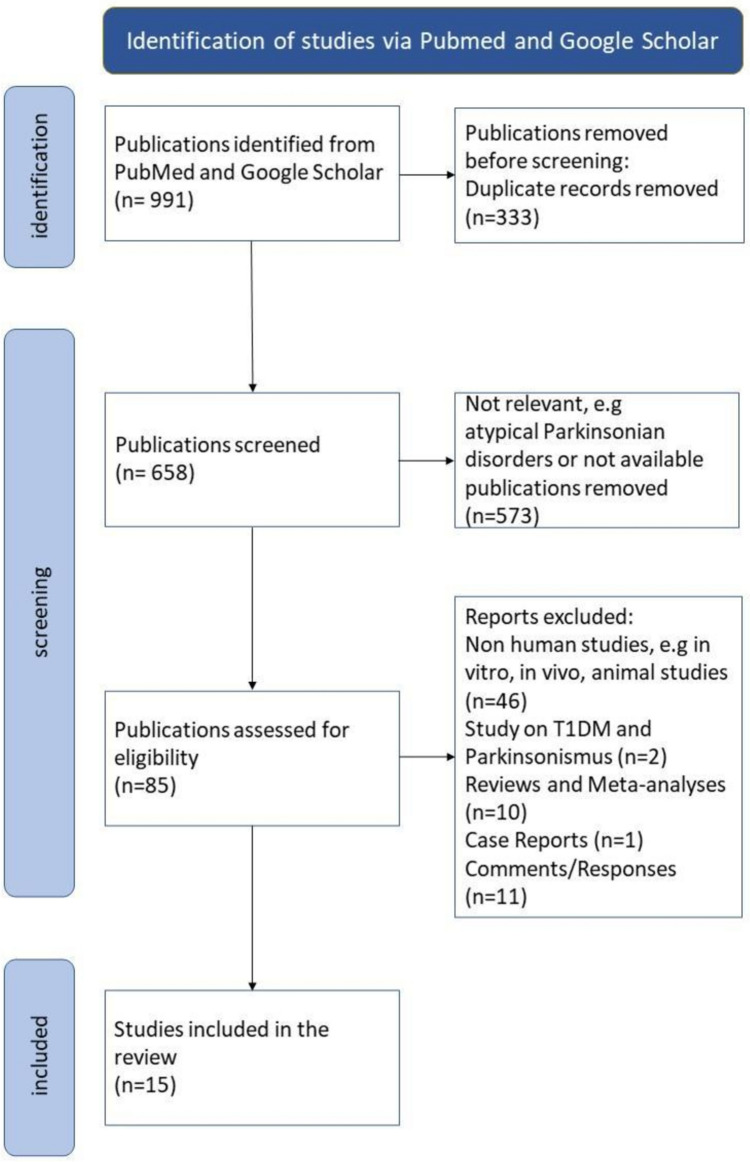
PRISM Flow Chart

Longitudinal cohort studies on the effect of type two diabetes on incidence of PD, neurological progression and PD biomarkers were searched via PubMed and Google Scholar (Fig. 1). Search terms were “Parkinson’s disease” And “Diabetes” And “T2D” And And “Levodopa”. Only studies with at least 10 PD patients per group and longitudinal data were included. Studies had to be published in English or German language before December 2023 for inclusion in this analysis.

### Exclusion criteria

Studies reporting data on atypical or undetermined Parkinsonian syndromes, and cell or animal studies were excluded. Studies with other specific forms of diabetes mellitus (DM), review articles and meta-analyses were also excluded.

### Data sharing statement

Data are available on reasonable request to corresponding author MK.

## Results

### Impact of diabetes mellitus type two on the incidence of Parkinson’s disease

As is shown in Table 1, in 6 out of 7 publications addressing the topic, T2DM was described as a risk factor for PD (Hu et al. 2007; Xu et al. 2011; Yang et al. 2017; De Pablo-Fernandez et al. 2018; Rhee et al. 2020; Gialluisi et al. 2023).

**Table 1:** Summary of studies that evaluate the association between T2DM and incidence of PD

References	Country	Sample size	Study design	HR/RR/OR	95% CI
Gialluisi et al J Neurol 2023	Italy	23,901	prospective cohort	HR = 1.85	1.24—2.76
Rhee et al. 2020	South Korea	15,168,021	prospective cohort	Duration of T2DM < 5 years: HR = 1.030 Duration of T2DM ≥ 5 years: HR = 1.618	Duration of T2DM < 5 years: 1.009—1.067 Duration of T2DM ≥ 5 years: 1.566—1.672
De Pablo-Fernandez et al. 2018	UK	2,017,115	retrospective cohort	HR = 1.32	1.29—1.35
Yang et al. 2017	China	145,176	prospective cohort	HR = 1.19	1.08—1.32
Xu et al. 2011	USA	288,662	Prospective cohort	OR = 1.41	1.20–1.66
Hu et al. 2007	Finland	51,552	Prospective cohort	HR = 1.85	1.23—2.80
Simon et al. 2007	USA	171,879	prospective cohort	RR = 1.04	0.74–1.46

*CI* confidence interval, *HR* Hazard ratio, *OR* odds ratio, *RR* relative ratio, *T2DM* diabetes mellitus type two

A population-based Korean cohort study demonstrated that T2DM as a comorbidity increased the risk of PD (Rhee et al. 2020). This study included 15,168,021 adults aged 40 years and older, divided into groups based on the presence of T2DM, impaired fasting glucose, duration of T2DM < 5 years, and duration of T2DM ≥ 5 years. During the observation period of 49,076,148.74 person-years, 31,577 patients developed PD. Compared to the group without DM or impaired fasting glucose, the hazard ratio (HR) was 1.03 (95% CI 1.009—1.067) in the group with T2DM and a duration < 5 years, and 1.618 (95% CI 1.566—1.672) in the group with T2DM and a duration ≥ 5 years, indicating that T2DM increased the risk of PD. Especially in those patients with longer duration of T2DM, the risk of PD significantly increased with the duration of diabetes.

In a Finnish study by Hu et al., including 51,552 men and women without a history of PD but with or without prior T2DM, 324 men and 309 women developed incident PD during a mean follow-up of 18.0 years. The HR for the occurrence of PD in patients with T2DM compared to those without T2DM was 1.85 (95% CI, 1.23—2.80) (Hu et al. 2007).

Gialluisi et al. examined various influencing factors on PD in a prospective study, including T2DM. Of the 23,901 individuals included, 213 developed incident PD during a median follow-up of 11.18 years. The risk of developing PD with comorbid T2DM in the multivariable Cox proportional hazards analysis yielded a HR of 1.85 (95% CI, 1.24—2.76) (Gialluisi et al. 2023).

Another prospective study from the USA investigated the association between self-reported T2DM and the future risk of PD in 288,662 participants. From 1995 to 2011, 1,565 participants developed PD. The risk of developing PD in patients with T2DM was derived from logistic regression models and showed an odds ratio (OR) of 1.41 (95% CI 1.20—1.66). Additionally, the risk was increased in individuals who had been diagnosed with T2DM for more than 10 years at baseline (OR = 1.75 (1.36—2.25) compared to those with a duration of less than 10 years (OR = 1.11 (0.89—1.38) (Xu et al. 2011).

De Pablo-Fernandez et al. analyzed data from a retrospective cohort study from 1999 to 2011 to estimate the risk of Parkinson's disease in patients with T2DM using Cox regression models. A total of 2,017,115 subjects with T2DM and 6,173,208 subjects without DM were included in the analysis. An increased risk of PD was observed in subjects with T2DM, with a HR of 1.32 (95% CI 1.29—1.35; p < 0.0001), compared to subjects without DM (De Pablo-Fernandez et al. 2018).

Yang et al. included a total of 36,294 patients with newly diagnosed T2DM and 108,882 individuals in the control group without DM between January 1, 2000, and December 31, 2006. Participants were followed until the first manifestation of PD or until December 31, 2011. The HR for the occurrence of PD in patients with pre-existing T2DM was estimated using the Cox proportional hazards regression model. Compared to the cohort without pre-existing DM, the incidence density of PD in the cohort with T2DM was 1.36 times higher (1.53 vs. 2.08 per 1000 person-years), with an HR of 1.19 (95% CI 1.08—1.32) adjusted for age, sex, comorbidities, and medication use (Yang et al. 2017).

A study by Simon et al. (Simon et al. 2007) reported no significant association between T2DM and the risk of developing PD. In this large prospective cohort study, using data from the Nurses' Health Study and the Health Professionals Follow-up Study, the relative risk (RR) was calculated as 1.04 (95% CI: 0.74–1.46). Participants, including 121,046 women and 50,833 men, were followed for a median duration of 22.9 years (women) and 12.6 years (men), with 530 cases of PD identified during the observation period. Exposures were assessed through self-reported doctor-diagnosed disease, rather than by direct clinical examination.

### Impact of type two diabetes mellitus on the clinical progression of Parkinson patients

#### Influence of type two diabetes mellitus on motor progression

Pagano et al. investigated the influence of T2DM on clinical progression in a case–control study. 25 patients with both T2DM and PD were compared with 25 patients with PD alone and 14 patients with T2DM alone. Additionally, the control group consisted of 14 patients without DM or PD. The clinical diagnosis of T2DM was confirmed by two consecutive fasting serum glucose measurements with values > 126 ml/dL. The endpoint for motor progression was defined as a change of one point on the Hoehn & Yahr scale. The cohorts were followed for 36 months with assessments conducted every 6 months. In the Cox proportional hazards analysis, the presence of T2DM in PD patients was associated with a higher risk of faster motor decline with an HR = 4.521 (95% CI 1.468 – 13.926; p < 0.01) (Pagano et al. 2018).

In the case–control study by Kotagal et al., 39 subjects were divided into two groups: 13 with PD and T2DM, and 26 with PD alone, matched for age, sex, disease duration, and comparable controls. All subjects underwent positron emission tomography to assess the volume ratio of [11C]dihydrotetrabenazine binding, a marker for nigrostriatal dopaminergic innervation, along with assessment of motor status using the Unified Parkinson's Disease Rating Scale (UPDRS). PD patients with comorbid T2DM showed increased postural instability compared to those without diabetes, and also exhibited more gait difficulties (p < 0.0005), although there was no significant difference of the mean striatal [11C]dihydrotetrabenazine distribution volume ratio between PD with T2DM and PD without DM (Kotagal et al. 2013).

In another case–control study, 89 patients with PD and comorbid T2DM were compared with 89 patients with PD alone, matched for gender, body mass index, and disease duration. Cereda et al. found that PD patients with comorbid T2DM had higher scores on the Unified Parkinson's Disease Rating Scale (UPDRS) for Part III (Motor Examination) (22.3 ± 9.0 vs. 19.3 ± 7.9; p < 0.019) and Part II (Motor Aspects of Experiences of Daily Living) (9.7 ± 5.1 vs. 8.3 ± 4.3; p < 0.049) compared to PD patients without DM (Cereda et al. 2012).

Ou et al. investigated in a prospective cohort study from March 2009 to August 2020 whether the control status of T2DM affects the progression of PD. 379 subjects were divided into three cohorts. 49 (12.9%) had both T2DM and PD. 22 (44.9%) of them had poorly controlled T2DM based on the HbA1c levels. Multivariate Cox proportional hazard regression models were used to assess predictors of PD progression. Rapid motor progression was defined as an increase of at least 14 points in the UPDRS-III score at a follow-up of 4.0 ± 2.4 years. In the cohort with poorly controlled T2DM, an increase in UPDRS-III scores ≥ 14 points was associated with an HR 2.060 (95% CI 1.165—3.641) compared to the control group without T2DM. In the cohort with well-controlled T2DM, the HR = 1.066 (95% CI 0.572—1.986) adjusted for age, sex, age at disease onset, body mass index, UPDRS-III scores, and Montreal Cognitive Assessment (MoCA) scores. Another endpoint was reaching Hoehn & Yahr stage ≥ 3. In the cohort with poorly controlled T2DM, the hazard ratio was 2.079 (95% CI 1.212 – 3.566) compared to an HR = 0.879 (95% CI 0.413 – 1.871) in the cohort with well-controlled T2DM (Ou et al. 2021).

In the study by Ogaki et al., the influence of diabetes mellitus on motor and non-motor symptoms in PD was investigated. In the cross-sectional study of 140 PD patients with available medical history and HbA1c serum levels, the correlation between HbA1c levels and clinical variables was analyzed. For this purpose, 23 PD patients with T2DM were grouped by severity of diabetes into complicated or uncomplicated DM based on HbA1c levels. The control group consisted of PD patients without DM. PD patients with T2DM had higher the Movement Disorder Society- Unified Parkinson's Disease Rating Scale Part III (MDS-UPDRS-III) scores of 36.3 ± 19.5 compared to MDS-UPDRS-III scores of 29.2 ± 15.3 in PD patients without DM. Additionally, PD patients with HbA1c levels > 5.7 exhibited elevated MDS-UPDRS-III scores of 33.3 ± 16.0 compared to PD patients with HbA1c levels ≤ 5.7 with MDS-UPDRS-III scores of 27.2 ± 15.6, indicating worse motor impairment in PD patients with higher HbA1c levels (Ogaki et al. 2023).

#### Influence of Diabetes mellitus type two on cognition

Pagano et al. also examined in the aforementioned study the influence of T2DM on cognition in the same cohorts. Subjectively reported memory problems of PD patients were assessed, along with six different neuropsychiatric tests (Letter-Number Sequencing Test, Semantic Fluency Test, Hopkins Verbal Learning Test-Revised Recall, Hopkins Verbal Learning Test-Revised Recognition Discrimination, Symbol Digit Modalities Test, and Benton Judgement of Line Orientation). Endpoints were defined as 2 out of 6 pathological neuropsychiatric tests and affirmation of memory problems. In the Cox proportional hazards analysis, the presence of T2DM in PD patients was associated with a risk of faster cognitive decline with an HR of 9.314 (95% CI 1.164—74.519; p < 0.05) (Pagano et al. 2018).

The research paper by Bohnen et al. also examined the effects of comorbid T2DM on cognition in patients with PD. In the cross-sectional study, 148 patients with PD, adjusted for age and Hoehn & Yahr stage, were divided into two cohorts. Fifteen PD patients had comorbid T2DM, while 133 patients did not. All subjects underwent [11 C] Methyl-4-Piperidinylpropionate (PMP)-Acetylcholinesterase (AChE)-PET imaging to evaluate cortical cholinergic denervation, [11C]dihydrotetrabenazine (DTBZ)-PET imaging to determine nigrostriatal denervation, and neuropsychological evaluations. A global cognitive Z-score was calculated based on normative data. Cognitive differences between patients with and without comorbid T2DM were assessed using analysis of covariance. The Z-score indicates whether the collected data are above or below the previously obtained average value. In the analyzed cohorts, PD patients with T2DM had a significantly lower mean global Z-score (−0.98 ± 1.01) compared to PD patients without DM (−0.36 ± 0.91; F = 7.76; p = 0.0061) p < 0.0001 (Bohnen et al. 2014).

The aforementioned study by Ogaki et al. also investigated the influences of T2DM on cognitive performance in PD patients. Binary logistic regression analysis, including age, sex, disease duration, MMSE scores (Mini-Mental State Examination), and MDS-UPDRS-III score as independent variables, revealed that a low MMSE score was an independent factor for PD patients with T2DM with an OR 0.823 (95% CI 0.714–0.930; p = 0.032) and PD patients with high HbA1c levels OR of 0.879 (95% CI 0.781–0.989; p = 0.032), meaning that a low MMSE score is associated with a higher risk of PD-DM (Ogaki et al. 2023).

Uyar et al. examined the association between HbA1c levels and cognitive impairment in 195 patients with PD and comorbid T2DM. The HbA1c levels of PD patients correlated significantly with the MoCA score. PD patients with comorbid T2DM had lower MoCA scores, indicating cognitive impairment (Uyar et al. 2022).

#### Influence of diabetes mellitus type two on treatment response

Cereda et al. investigated whether there were differences in levodopa treatment doses in PD patients with comorbid T2DM compared to PD patients without. 89 patients with newly diagnosed PD and pre-existing T2DM were included from 2007 to 2010. The control group consisted of 89 PD patients without T2DM, matched by gender, body mass index (± 1 kg/m^2^), and disease duration (± 1 year). PD patients with comorbid T2DM had higher levodopa treatment doses than PD patients without DM (mg/day, 448 ± 265 vs. 300 ± 213; p < 0.0001; mg/kg/day, 5.8 ± 4.0 vs. 3.8 ± 2.9; p < 0.0001) (Cereda et al. 2012).

#### Influence of diabetes mellitus type two on overall mortality

Pezzoli et al. examined the influence of antidiabetic therapy on the age at onset of PD and its impact on overall mortality. Data from 8,380 patients were included. There was a delayed onset of PD by 6.2 years (p < 0.001) in patients receiving antidiabetic therapy for T2DM before the onset of PD compared with patients in whom T2DM occurred after the onset of PD and those without DM. Pre-existing T2DM also showed a negative effect on prognosis with an adjusted hazard ratio of 1.64 (95% CI 1.33—2.02; p < 0.0019). In patients who developed DM after the onset of PD, there was no negative impact on survival HR = 0.86 (95% CI 0.53 – 1.39; p = 0.54) (Pezzoli et al. 2023).

### Impact of diabetes mellitus type two on the biomarker profile of Parkinson patients

In the case–control study by Pagano et al., the influence of T2DM on neurological progression also included the impact on tau protein levels in cerebrospinal fluid in 25 patients with T2DM and PD, 25 patients with PD without DM, and 14 patients with T2DM alone. The control group consisted of 14 subjects without PD or DM. The samples from PD patients with T2DM showed higher pTau181 and total Tau level in cerebrospinal fluid (p < 0.05) compared to the other cohorts (Pagano et al. 2018). Additionally, Pagano et al.'s study showed that PD patients with T2DM exhibited lower striatal dopamine transporter binding on [123I]FP-CIT SPECT imaging compared to Parkinson's patients without DM (Pagano et al. 2018).

In the study by Uyar et al. with 195 subjects, T2DM in PD patients was associated with higher serum neurofilament light chain levels in the linear regression model after adjustment for age and BMI, serving as a marker for more profound neuronal damage in these patients (Uyar et al. 2022).

## Discussion

In summary, these studies show that T2DM may not only adversely affect the incidence of PD, but may also harmfully contribute to motor progression, cognitive function, treatment response and overall mortality in PD patients. Further, T2DM is associated with changes in the biomarker profile of PD patients, including changes in cerebrospinal fluid tau and serum neurofilament light chain levels, as well as reduced striatal dopamine transporter binding on dopaminergic SPECT imaging.

While six of the seven selected studies indicated that T2DM is a risk factor for the development of PD, one study (Simon et al. 2007) drew a different conclusion. In this study, the diagnosis of PD and risk factors, including T2DM, were assessed through self-reported questionnaires. Potential misclassification of exposures cannot be ruled out, and such misclassification might dilute or mask any true associations between T2DM and PD. However, this self-report method was validated and used in many other studies, including the paper by Xu et al., which showed a positive association between PD and T2DM (Xu et al. 2011). Additionally, the Simon et al. study included women aged 30–55 years and men aged 40–75 years at baseline without any further stratification. The inclusion of younger participants may have captured individuals before diabetes-related neurodegenerative changes had fully developed. In contrast, the study by Rhee also included a few of participants as young as 40 and further stratified individuals by disease duration. It turned out that the risk of PD increases with the duration of T2DM (Rhee et al. 2020). Moreover, the Simon et al. study included participants in the Nurses’ Health Study and Health Professionals Follow-up Study. They were predominantly health-conscious professionals, which may limit the generalizability of the findings.

The mechanisms of the negative influence of T2DM on PD are not yet clear. T2DM is a chronic metabolic disorder with hyperglycemia, caused by defective insulin secretion by pancreatic β-cells and the inability of insulin-sensitive tissues to respond appropriately to insulin (Stumvoll et al. 2005). It was assumed that elevated blood sugar levels can lead to the formation of advanced glycation end-products (AGEs), which could promote the aggregation of proteins like α-synuclein and tau (Sirangelo and Iannuzzi 2021; Lv et al. 2022; Aguirre-Vidal et al. 2024). Moreover, methylglyoxal, a byproduct of glycolysis, can react with dopamine to form a neurotoxin called ADTIQ (1-acetyl-6,7-dihydroxy-1,2,3,4-tetrahydroisoquinoline) (Stumvoll et al. 2005), which specifically targets dopaminergic neurons (Deng et al. 2012). On the other side, insulin resistance impairs the PI3K/AKT signaling pathway, leading to the activation of GSK-3β and NF-κB and further resulting in increased apoptosis, autophagy, oxidative stress, and mitochondrial dysfunction (Ruiz-Pozo et al. 2023). The insulin resistance could also decrease dopamine turnover and reduce expression of dopamine transporters in the striatum (Kleinridders et al. 2015). This assumption is consistent with the above-mentioned observation, that PD patients with T2DM exhibited reduced striatal dopamine transporter binding(Pagano et al. 2018). Actually, it is suggested that insulin resistance present in the brain, can damage and increase the permeability of the blood–brain barrier, exacerbate neuroinflammation, and is a common feature of neurodegenerative diseases (Kopp et al. 2022). Furthermore, it was shown that the presence of the islet amyloid polypeptide (IAPP) in T2DM patients can accelerate the aggregation of α-synuclein, tau as well as other prion-like proteins through cross-talk (Horvath and Wittung-Stafshede 2016; Denroche and Verchere 2018; Mucibabic et al. 2020). As mentioned above, PD patients with T2DM also showed higher tTau and pTau181 concentrations in the cerebrospinal fluid (Pagano et al. 2018).

On the contrary, a couple of studies suggested that DM type 1 (T1DM) may have a protective effect on PD risk, as both types of diabetes share hyperglycemia (Geng et al. 2024; Mai et al. 2024). Different from T2DM, which occurs due to insulin resistance and shares a close link with metabolic syndrome, T1DM is caused by the autoimmune destruction of insulin-secreting pancreatic islet beta cells (Eizirik et al. 2020). Moreover, aggregates of islet amyloid polypeptide (IAPP), which were identified in T2DM, have not been implicated in T1DM (Denroche and Verchere 2018). We propose that the increased susceptibility of T2D patients to developing PD may not be primarily due to hyperglycemia, but rather due to insulin resistance or the interaction between IAPP and α-synuclein and/or tau.

Notably, it could be possible that the interaction between T2DM and PD could not only mean that T2DM may trigger PD, but also PD triggering T2DM. It was also found that α-synuclein promotes IAPP fibril formation in vitro and β-cell formation in the pancreas of mice (Mucibabic et al. 2020).

Considering T2DM as an established risk factor for PD, antidiabetic therapy would be a therapeutic strategy for PD. Biguanide (metformin), dipeptidyl peptidase-4 (DPP-4) inhibitors, alogliptin, evogliptin, gemigliptin, linagliptin, saxagliptin, sitagliptin, teneligliptin, vildagliptin), thiazolidinediones (rosiglitazone, pioglitazone), Glucagon-like peptide-1 (GLP-1) receptor agonists (albiglutide, dulaglutide, exenatide, liraglutide, lixisenatide, semaglutide, tirzepatide), Sodium-glucose cotransporter 2 (SGLT-2) inhibitors (dapagliflozin, empagliflozin, ertugliflozin, canagliflozin, sotagliflozin) and insulin are extensively used for the management of T2DM.

GLP-1 is an incretin hormone that helps maintain glucose balance by stimulating insulin secretion and production while suppressing glucagon release (Nadkarni et al. 2014). Recently, Lixisenatide, a GLP1 receptor agonist, showed a neuroprotective effect in a phase 2, double-blind, randomized, placebo-controlled trial with 156 PD patients (Meissner et al. 2024). Similar trials with GLP-1 receptor agonists exenatide (Athauda et al. 2017) and NLY01, a brain-penetrant, pegylated, longer-lasting version of exenatide (McGarry et al. 2024) also showed positive effects on motor symptoms of PD patients. Some studies showed that PD patients with T2DM who were treated with the GLP-1 receptor agonist liraglutide improved non-motor symptoms (Nadkarni et al. 2014; Malatt et al. 2022). Besides GLP1 receptor agonist, other oral glucose lowering drugs such as metformin, thiazolidinediones and DPP-4 inhibitors, as well as insulin have been tested in preclinical models or in small clinical studies and proven to be effective in the treatment of PD (Pang et al. 2016; Svenningsson et al. 2016; Novak et al. 2019; Brauer et al. 2020; Wang et al. 2024).

Similar to the hypothesis that the increased susceptibility of T2DM patients to developing PD may not be primarily driven by hyperglycemia, we propose that the mechanisms underlying the protective benefits of antidiabetic drugs in reducing the risk of PD may stem from mechanisms beyond glucose control. Studies have shown that using DPP4 inhibitors or GLP-1 mimetics is associated with a 36–60% lower risk of PD compared to other oral antidiabetic drugs, suggesting different therapeutic efficacy among different antidiabetic drugs (Brauer et al. 2020). Additionally, a placebo-controlled study demonstrated that intranasal insulin improves cognitive and motor function in PD patients without significantly affecting blood glucose levels, further indicating that these benefits may occur independently of glucose regulation (Novak et al. 2019).

Instead, the neuroprotective effect of these antidiabetic medications may be related to their anti-apoptotic, anti-oxidative, neurotrophic and anti-inflammatory mechanisms. For example, GLP-1 treatment was found to be neuroprotective in an in-vitro PD model through increasing PKA and PI3K pathway activity, reducing expression of apoptotic factors, and elevating expression of anti-apoptotic factors (Kopp et al. 2022). Metformin was shown to delay astrocyte senescence via inhibiting astrocytic Mfn2-cGAS activation in vitro and in a mouse model of PD (Wang et al. 2024). Insulin was found to induce anti-inflammatory phenotypes in reactive glia through activation of the P13K/Akt (Pang et al. 2016). Unfortunately, there are few studies of anti-diabetic therapy targeting other mechanisms, such as the insulin resistance-related decrease in dopamine turnover and the cross-talk between IAPP and α-synuclein.

From a clinical perspective T2DM appears to be a critical factor influencing the progression and the prognosis of PD. Thus, early screening for T2DM in PD patients is essential (Klietz et al. 2019). Identifying and addressing comorbid T2DM may not only prevent or delay the development of DM-related complications, but also the PD related motor and cognitive decline.

The connection between T2DM and PD highlights the importance of developing tailored treatment strategies for PD patients with T2DM. The presence of T2DM may affect the efficacy of dopaminergic treatments, including the levodopa challenge, emphasizing the need for careful selection of therapeutic options. For instance, a PD patient with T2DM who shows a limited benefit from a dopaminergic treatment, still may respond well to advanced therapies, such as deep brain stimulation or pump therapies. Similarly, when considering treatment for T2DM in PD patients, it is crucial to choose antidiabetic medications that offer dual benefits for both the metabolic and neurological disease. For example, it was shown that GLP-1 receptor agonists not only manage hyperglycemia but may also exhibit potential neuroprotective effects(Meissner et al. 2024). It would also be very interesting to study if such neuroprotective effects persist also in PD patients with T2DM who have other metabolic risk factors, such as obesity.

Furthermore, given the potential neuroprotective effect of a couple of antidiabetic medications, it is valuable to investigate if the neuroprotective effect extends to PD patients without DM. If such medications are beneficial only for a subset of PD patients without T2DM, it is crucial to identify the determining factor that influences their efficacy.

### Limitations

In this systematic review only longitudinal PD patient cohorts were included; however, no interventional trials were included in this analysis. Further, treatment of diabetes in these cohorts was rarely reported systematically and was not available for systematic analysis. Because of this limitation no specific recommendations for treatment of diabetes in PD could be drawn from this systematic review.

## Conclusion

T2DM has a profound impact on patients with PD. The incidence of PD is significantly raised in T2DM patients. Further, T2DM PD patients will suffer from faster motor and cognitive decline. Biomarkers of neurodegenerative diseases like neurofilament light, tau and amyloid beta were all regulated in an undesirable direction in T2DM patients. Physicians should screen PD patients for diabetes and refer them for early T2DM treatment initiation. Further studies are needed to elucidate shared disease mechanisms and possible treatment strategies.

## Data Availability

Data are available on reasonable request to corresponding author MK.

## References

[CR1] Aguirre-Vidal Y, Montes S, Mota-López AC, Navarrete-Vázquez G (2024) Antidiabetic drugs in Parkinson’s disease. Clin Park Relat Disord 11:100265 39149559 10.1016/j.prdoa.2024.100265 PMC11325349

[CR2] Athauda D, Maclagan K, Skene SS et al (2017) Exenatide once weekly versus placebo in Parkinson’s disease: a randomised, double-blind, placebo-controlled trial. Lancet 390:1664–1675 28781108 10.1016/S0140-6736(17)31585-4 PMC5831666

[CR3] Bohnen NI, Kotagal V, Müller MLTM et al (2014) Diabetes mellitus is independently associated with more severe cognitive impairment in Parkinson disease. Parkinsonism Relat Disord 20:1394–1398 25454317 10.1016/j.parkreldis.2014.10.008 PMC4314515

[CR4] Brauer R, Wei L, Ma T et al (2020) Diabetes medications and risk of Parkinson’s disease: a cohort study of patients with diabetes. Brain 143:3067–3076 33011770 10.1093/brain/awaa262 PMC7794498

[CR5] Cereda E, Barichella M, Cassani E et al (2012) Clinical features of Parkinson disease when onset of diabetes came first: A case-control study. Neurology 78:1507–1511 22539572 10.1212/WNL.0b013e3182553cc9

[CR6] De Pablo-Fernandez E, Goldacre R, Pakpoor J et al (2018) Association between diabetes and subsequent Parkinson disease: A record-linkage cohort study. Neurology 91:e139–e142 29898968 10.1212/WNL.0000000000005771

[CR7] Deng Y, Zhang Y, Li Y et al (2012) Occurrence and distribution of salsolinol-like compound, 1-acetyl-6,7-dihydroxy-1,2,3,4-tetrahydroisoquinoline (ADTIQ) in parkinsonian brains. J Neural Transm 119:435–441 22065205 10.1007/s00702-011-0724-4

[CR8] Denroche HC, Verchere CB (2018) IAPP and type 1 diabetes: implications for immunity, metabolism and islet transplants. J Mol Endocrinol 60:R57–R75 29378867 10.1530/JME-17-0138

[CR9] Eizirik DL, Pasquali L, Cnop M (2020) Pancreatic β-cells in type 1 and type 2 diabetes mellitus: different pathways to failure. Nat Rev Endocrinol 16:349–362 32398822 10.1038/s41574-020-0355-7

[CR10] Geng C, Meng K, Zhao B et al (2024) Causal relationships between type 1 diabetes mellitus and Alzheimer’s disease and Parkinson’s disease: a bidirectional two-sample Mendelian randomization study. Eur J Med Res 29:53 38229119 10.1186/s40001-023-01628-z PMC10790511

[CR11] Gialluisi A, De Bartolo MI, Costanzo S et al (2023) Risk and protective factors in Parkinson’s disease: a simultaneous and prospective study with classical statistical and novel machine learning models. J Neurol 270:4487–4497 37294324 10.1007/s00415-023-11803-1

[CR12] Greten S, Müller-Funogea JI, Wegner F et al (2021) Drug safety profiles in geriatric patients with Parkinson’s disease using the FORTA (Fit fOR The Aged) classification: results from a mono-centric retrospective analysis. J Neural Transm (Vienna) 128:49–60 33263172 10.1007/s00702-020-02276-x PMC7815558

[CR13] Horvath I, Wittung-Stafshede P (2016) Cross-talk between amyloidogenic proteins in type-2 diabetes and Parkinson’s disease. Proc Natl Acad Sci U S A 113:12473–12477 27791129 10.1073/pnas.1610371113 PMC5098634

[CR14] Hu G, Jousilahti P, Bidel S et al (2007) Type 2 diabetes and the risk of Parkinson’s disease. Diabetes Care 30:842–847 17251276 10.2337/dc06-2011

[CR15] Kleinridders A, Cai W, Cappellucci L et al (2015) Insulin resistance in brain alters dopamine turnover and causes behavioral disorders. Proc Natl Acad Sci U S A 112:3463–3468 25733901 10.1073/pnas.1500877112 PMC4371978

[CR16] Klietz M, Greten S, Wegner F, Höglinger GU (2019) Safety and tolerability of pharmacotherapies for Parkinson’s disease in geriatric patients. Drugs Aging 36:511–530 30937878 10.1007/s40266-019-00654-z

[CR17] König A, Outeiro TF (2024) Diabetes and Parkinson’s Disease: understanding shared molecular mechanisms. J Parkinsons Dis 14:917–924 38995799 10.3233/JPD-230104 PMC11307096

[CR18] Kopp KO, Glotfelty EJ, Li Y, Greig NH (2022) Glucagon-like peptide-1 (GLP-1) receptor agonists and neuroinflammation: implications for neurodegenerative disease treatment. Pharmacol Res 186:106550 36372278 10.1016/j.phrs.2022.106550 PMC9712272

[CR19] Kotagal V, Albin RL, Müller MLTM et al (2013) Diabetes is associated with postural instability and gait difficulty in Parkinson disease. Parkinsonism Relat Disord 19:522–526 23462483 10.1016/j.parkreldis.2013.01.016 PMC3607954

[CR20] Lv Y-Q, Yuan L, Sun Y et al (2022) Long-term hyperglycemia aggravates α-synuclein aggregation and dopaminergic neuronal loss in a Parkinson’s disease mouse model. Transl Neurodegener 11:14 35255986 10.1186/s40035-022-00288-z PMC8900445

[CR21] Mai AS, Tan BJ-W, Sun Q-Y, Tan E-K (2024) Association between Type 1 Diabetes Mellitus and Parkinson’s Disease: a mendelian randomization study. J Clin Med Res 13(2):561. 10.3390/jcm13020561 10.3390/jcm13020561 PMC10816052 38256693

[CR22] Malatt C, Wu T, Bresee C et al (2022) Liraglutide Improves Non-Motor Function and Activities of Daily Living in Patients with Parkinson’s disease: A Randomized, Double-Blind, Placebo-Controlled Trial (P9–11.005). Neurology 98(18_Supplement):3068

[CR23] McGarry A, Rosanbalm S, Leinonen M et al (2024) Safety, tolerability, and efficacy of NLY01 in early untreated Parkinson’s disease: a randomised, double-blind, placebo-controlled trial. Lancet Neurol 23:37–45 38101901 10.1016/S1474-4422(23)00378-2

[CR24] Meissner WG, Remy P, Giordana C et al (2024) Trial of Lixisenatide in Early Parkinson’s Disease. N Engl J Med 390:1176–1185 38598572 10.1056/NEJMoa2312323

[CR25] Mucibabic M, Steneberg P, Lidh E et al (2020) α-Synuclein promotes IAPP fibril formation in vitro and β-cell amyloid formation in vivo in mice. Sci Rep 10:20438 33235246 10.1038/s41598-020-77409-z PMC7686322

[CR26] Nadkarni P, Chepurny OG, Holz GG (2014) Regulation of glucose homeostasis by GLP-1. Prog Mol Biol Transl Sci 121:23–65 24373234 10.1016/B978-0-12-800101-1.00002-8 PMC4159612

[CR27] Novak P, Pimentel Maldonado DA, Novak V (2019) Safety and preliminary efficacy of intranasal insulin for cognitive impairment in Parkinson disease and multiple system atrophy: A double-blinded placebo-controlled pilot study. PLoS ONE 14:e0214364 31022213 10.1371/journal.pone.0214364 PMC6483338

[CR28] Ogaki K, Fujita H, Nozawa N et al (2023) Impact of diabetes and glycated hemoglobin level on the clinical manifestations of Parkinson’s disease. J Neurol Sci 454:120851 37931442 10.1016/j.jns.2023.120851

[CR29] Ou R, Wei Q, Hou Y et al (2021) Effect of diabetes control status on the progression of Parkinson’s disease: A prospective study. Ann Clin Transl Neurol 8:887–897 33764699 10.1002/acn3.51343 PMC8045952

[CR30] Pagano G, Polychronis S, Wilson H et al (2018) Diabetes mellitus and Parkinson disease. Neurology 90:e1654–e1662 29626177 10.1212/WNL.0000000000005475

[CR31] Pang Y, Lin S, Wright C et al (2016) Intranasal insulin protects against substantia nigra dopaminergic neuronal loss and alleviates motor deficits induced by 6-OHDA in rats. Neuroscience 318:157–165 26777890 10.1016/j.neuroscience.2016.01.020 PMC4753102

[CR32] Pezzoli G, Cereda E, Amami P et al (2023) Onset and mortality of Parkinson’s disease in relation to type II diabetes. J Neurol 270:1564–1572 36436068 10.1007/s00415-022-11496-y PMC9971073

[CR33] Rhee SY, Han K-D, Kwon H et al (2020) Association Between Glycemic Status and the Risk of Parkinson Disease: a Nationwide Population-Based Study. Diabetes Care 43:2169–2175 32611610 10.2337/dc19-0760 PMC7440896

[CR34] Ruiz-Pozo VA, Tamayo-Trujillo R, Cadena-Ullauri S et al (2023) The Molecular Mechanisms of the Relationship between Insulin Resistance and Parkinson’s Disease Pathogenesis. Nutrients 15(16):3585. 10.3390/nu15163585 37630775 10.3390/nu15163585 PMC10458139

[CR35] Simon KC, Chen H, Schwarzschild M, Ascherio A (2007) Hypertension, hypercholesterolemia, diabetes, and risk of Parkinson disease. Neurology 69:1688–1695 17761552 10.1212/01.wnl.0000271883.45010.8a PMC2391077

[CR36] Sirangelo I, Iannuzzi C (2021) Understanding the Role of Protein Glycation in the Amyloid Aggregation Process. Int J Mol Sci 22(12):6609. 10.3390/ijms22126609 34205510 10.3390/ijms22126609 PMC8235188

[CR37] Stumvoll M, Goldstein BJ, van Haeften TW (2005) Type 2 diabetes: principles of pathogenesis and therapy. Lancet 365:1333–1346 15823385 10.1016/S0140-6736(05)61032-X

[CR38] Svenningsson P, Wirdefeldt K, Yin L et al (2016) Reduced incidence of Parkinson’s disease after dipeptidyl peptidase-4 inhibitors-A nationwide case-control study. Mov Disord 31:1422–1423 27431803 10.1002/mds.26734

[CR39] Uyar M, Lezius S, Buhmann C et al (2022) Diabetes, Glycated Hemoglobin (HbA1c), and Neuroaxonal Damage in Parkinson’s Disease (MARK-PD Study). Mov Disord 37:1299–1304 35384057 10.1002/mds.29009

[CR40] Veith Sanches L, Greten S, Doll-Lee J et al (2024) SEND-PD in Parkinsonian Syndromes: Results of a Monocentric Cross-Sectional Study. Neuropsychiatr Dis Treat 20:1849–1859 39372876 10.2147/NDT.S474584 PMC11453152

[CR41] von Eichel H, Heine J, Wegner F et al (2022) Neuropsychiatric symptoms in parkinson’s disease patients are associated with reduced health-related quality of life and increased caregiver burden. Brain Sci 12:89. 10.3390/brainsci12010089 35053832 10.3390/brainsci12010089 PMC8774188

[CR42] Wang M, Tian T, Zhou H et al (2024) Metformin normalizes mitochondrial function to delay astrocyte senescence in a mouse model of Parkinson’s disease through Mfn2-cGAS signaling. J Neuroinflammation 21:81 38566081 10.1186/s12974-024-03072-0 PMC10986112

[CR43] Xu Q, Park Y, Huang X et al (2011) Diabetes and risk of Parkinson’s disease. Diabetes Care 34:910–915 21378214 10.2337/dc10-1922 PMC3064050

[CR44] Yang Y-W, Hsieh T-F, Li C-I et al (2017) Increased risk of Parkinson disease with diabetes mellitus in a population-based study. Medicine (Baltimore) 96:e5921 28099356 10.1097/MD.0000000000005921 PMC5279101

